# Imaging Cancer Metabolism: Underlying Biology and Emerging Strategies

**DOI:** 10.2967/jnumed.117.199869

**Published:** 2018-09

**Authors:** Austin R. Pantel, Daniel Ackerman, Seung-Cheol Lee, David A. Mankoff, Terence P. Gade

**Affiliations:** 1 Department of Radiology, University of Pennsylvania, Philadelphia, Pennsylvania; 2 Penn Image-Guided Interventions Laboratory, Department of Radiology, University of Pennsylvania, Philadelphia, Pennsylvania; and; 3 Department of Cancer Biology, University of Pennsylvania, Philadelphia, Pennsylvania

**Keywords:** cancer metabolism, magnetic resonance imaging, molecular imaging, positron-emission tomography

## Abstract

Dysregulated cellular metabolism is a characteristic feature of malignancy that has been exploited for both imaging and targeted therapy. With regard to imaging, deranged glucose metabolism has been leveraged using ^18^F-FDG PET. Metabolic imaging with ^18^F-FDG, however, probes only the early steps of glycolysis; the complexities of metabolism beyond these early steps in this single pathway are not directly captured. New imaging technologies—both PET with novel radiotracers and MR-based methods—provide unique opportunities to investigate other aspects of cellular metabolism and expand the metabolic imaging armamentarium. This review will discuss the underlying biology of metabolic dysregulation in cancer, focusing on glucose, glutamine, and acetate metabolism. Novel imaging strategies will be discussed within this biologic framework, highlighting particular strengths and limitations of each technique. Emphasis is placed on the role that combining modalities will play in enabling multiparametric imaging to fully characterize tumor biology to better inform treatment.

**NOTEWORTHY**

- As a hallmark of the cancer phenotype, aberrant cancer metabolism has been clinically imaged with ^18^F-FDG PET, which probes tumor glycolysis.
- While an elevated glycolytic rate is a common feature of dysregulated metabolism in cancer, further advances in our understanding of cancer metabolism are providing unique opportunities for the development of clinically relevant imaging strategies.
- Novel PET- and MR-based biomarkers of cancer metabolism may be used independently, or in combination, to probe unique aspects of cancer metabolism and are being translated into the clinic.

Aberrant cellular metabolism has long been recognized as a primary feature of cancer. Nearly a hundred years ago, Otto Warburg described the propensity of malignant cells to rapidly metabolize glucose to lactate, a seemingly wasteful process in terms of net energy production (1). This effect, which now bears his name, has been extensively studied with increasing research interest and effort (2). In fact, the reprogramming of energy metabolism has recently been recognized as a hallmark of cancer (3). Dysregulated cellular metabolism in cancer, though, extends beyond glycolysis; complex interrelationships exist between energy catabolism and biosynthetic pathways (Fig. 1 (4)), as well as adaptive responses to oncogenic stress. Carbohydrate, amino acid, and lipid metabolism become similarly reprogrammed, providing opportunities for both targeted imaging and therapy. Indeed, Warburg’s fundamental discovery of deranged carbohydrate metabolism in cancer has been successfully imaged with the glucose analog ^18^F-FDG. PET imaging with ^18^F-FDG has gained widespread clinical acceptance as a marker of tumor glycolysis. ^18^F-FDG PET is currently approved by the Centers for Medicare and Medicaid services for numerous indications, including characterization, diagnosis, staging, and restaging of multiple malignancies (National Coverage Determination for PET Scans [220.6]).

**FIGURE 1.**
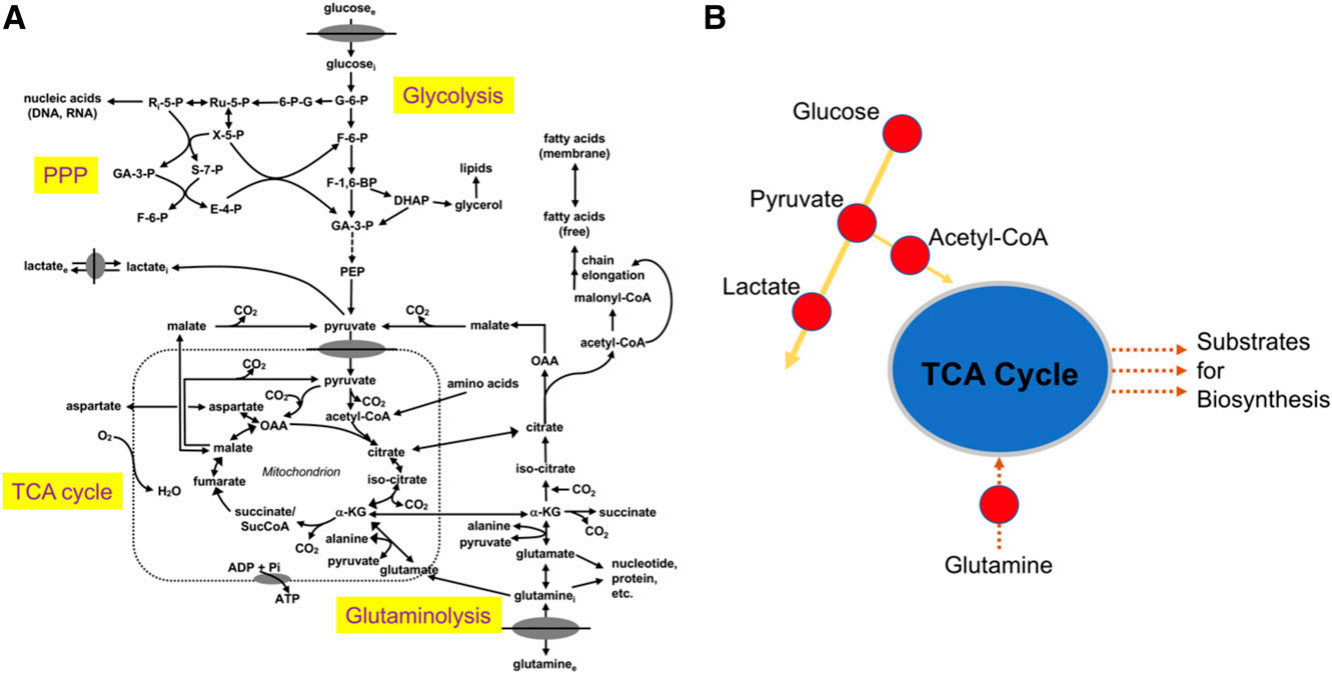
Simplified schematic of central cellular metabolic pathways (A) emphasizing key branch points and imaging targets (B). glucose_e_ = extracellular glucose; glucose_i_ = intracellular glucose; G-6-P = glucose-6-phosphate; 6-P-G = 6-phosphogluconate; Ru-5-P = ribulose-5-phosphate; Ri-5-P = ribose-5-phosphate; X-5-P = xylulose 5-phosphate; S-7-P = sedoheptulose 7-phosphate; GA-3-P = glyceraldehyde 3-phosphate; F-6-P = fructose 6-phosphate; E-4-P = erythrose 4-phosphate; PPP = pentose phosphate pathway; F-1,6-BP = fructose 1,6-bisphosphate; DHAP = dihydroxyacetone phosphate; PEP = phosphoenolpyruvate; lactate_i_ = intracellular lactate; lactate_e_ = extracellular lactate; CO_2_ = carbon dioxide; α-KG = α-ketoglutarate; OAA = oxaloacetate; O_2_ = oxygen; ADP = adenosine diphosphate; Pi = inorganic phosphate; glutamine_e_ = extracellular glutamine; glutamine_i_ = intracellular glutamine. (Adapted and reprinted with permission of (4).)

Despite its central role in clinical oncology, ^18^F-FDG PET provides a limited index of cancer metabolism because it probes only a single aspect of cellular metabolism. Given the complex interrelationships between numerous cellular pathways that have recently been elucidated, there is a renewed interest in the development of other targeted PET radiotracers. Moreover, given some inherent limitations of PET (e.g., limited capability for simultaneous imaging of multiple metabolic pathways), other imaging modalities have been studied and advanced for imaging cancer metabolism. Hyperpolarized MR spectroscopic imaging (MRSI), thermal polarization MR spectroscopy (MRS), and chemical exchange saturation transfer (CEST) MRI all offer unique capabilities and can provide new insights into cancer biology that cannot be obtained with PET imaging alone. With the introduction of hybrid imaging using PET/MRI scanners, these strategies can be combined to enable complementary and unique insights into cancer metabolism.

Recent technologic advances have facilitated a more detailed description of the intricate interrelationships and adaptations that characterize cancer metabolism. Such complexity underscores the fundamental challenges associated with imaging a dynamic biologic system. This review will build a framework to define these challenges by focusing on glucose metabolism as a model system, with a synopsis of the current state of imaging alternative metabolic pathways, specifically glutamine and acetate metabolism. Both PET and non-PET molecular modalities will be discussed, with particular attention to those with impending clinical applications. The integration of these complementary approaches with advances in our understanding of cancer biology may more completely characterize tumor metabolism to better direct and monitor the response to targeted cancer therapies. Numerous targeted therapies have recently advanced into the clinic; however, the development of targeted imaging agents that directly assess on-target effects has lagged. The development of such correlative imaging methods will allow a more tailored approach to cancer treatment with important implications for the advancement of current health-care initiatives, including precision medicine.

## MECHANISMS OF CELLULAR METABOLIC DYSREGULATION

The field of cancer metabolism has been reinvigorated over the past decade with transformative advances in our understanding of the biology underlying altered glucose metabolism. The fundamental role of oncogenes and tumor suppressor genes in effecting these alterations has been established, with such genetic alterations now known to coordinate a complex rewiring of cellular metabolism. For example, the serine/threonine kinase AKT, activated at the cell membrane by phosphatidylinositol 3-kinase, promotes localization of the glucose transporter 1 (Glut1) to the cell surface and increases hexokinase activity, both of which support increased glucose metabolism (5). The oncogenes KRAS and BRAF are known to increase Glut1 transcript expression and protein levels in colorectal cancer cell lines, with a resultant increase in glucose uptake and lactate formation (6). The c-Myc transcription factor, encoded by the c-Myc oncogene, increases lactate dehydrogenase-A expression through a direct interaction with its promoter. The lactate dehydrogenase-A enzyme converts pyruvate to lactate, providing a molecular mechanism for the final step of the Warburg hypothesis (7). In addition to genetic mutations, the tumor microenvironment plays an important role in modulating gene expression, emphasizing context-dependent metabolic alterations. In response to low oxygen tension, the transcription factor hypoxia-inducible factor 1α drives the expression of several target genes involved in glucose transport and glycolysis, including the Glut1 transporter (8,9). Hypoxia-inducible factor 1 directs pyruvate, the end product of glycolysis, away from the tricarboxylic acid cycle (TCA cycle) through transcriptional activation of lactate dehydrogenase-A and pyruvate dehydrogenase kinase 1. Pyruvate dehydrogenase kinase 1 inactivates pyruvate dehydrogenase to mitigate the conversion of pyruvate to acetyl–coenzyme A (CoA) (10). These alterations in glucose transport and gene expression exemplify elements of a concerted program of glycolytic flux modulation leading to the generation of lactate and, in turn, suggesting the importance of alternative fuels to maintain other necessary cellular pathways.

Although many molecular mechanisms of aerobic glycolysis have been elucidated, the fundamental benefit of the Warburg effect for the cancer phenotype remains uncertain. This uncertainty has centered primarily on the bioenergetic inefficiency of aerobic glycolysis, a seemingly wasteful process in terms of adenosine triphosphate (ATP) production (∼36 mol ATP/mol glucose through oxidative phosphorylation vs. ∼4 mol ATP/mol glucose through glycolysis) (11). Certainly, this inefficiency contrasts with the relative efficiency of cancer cells in modulating other metabolic processes under conditions of nutrient deprivation. Under nutrient-limiting conditions, cancer cells undergo metabolic reprogramming to scavenge proteins and lipids through activation of autophagy and macropinocytosis (12,13). Recent data support 2 compelling explanations for the persistence of this inefficient process in cancer cells. The first emphasizes the importance of accumulating glycolytic intermediates to facilitate biosynthesis, such that, if resources are plentiful and ATP is not a limiting factor in cellular proliferation, the bioenergetically inefficient use of the glucose carbon skeleton may not be disadvantageous. The enhanced flux of glucose to lactate may provide a selective advantage by facilitating the siphoning of biosynthetic intermediates and cofactors that are required for the unrestrained cellular proliferation that is perhaps the best-known hallmark feature of cancer (2,11,14). Among these, the production of the reducing equivalent nicotinamide adenine dinucleotide phosphate, an essential cofactor for anabolism, has been postulated as a benefit of the Warburg effect, with the pentose phosphate shunt producing 2 nicotinamide adenine dinucleotide phosphates for each glucose-6-phosphate (11). Indeed, expression of the tumor suppressor p53 has been shown to inactivate the rate-limiting step of the pentose phosphate shunt, glucose-6-phosphate dehydrogenase, with p53 mutants demonstrating increased pathway flux (15). Nonetheless, the importance of the pentose phosphate pathway has been debated as a sufficient source of nicotinamide adenine dinucleotide phosphate (16). The second explanation for aerobic glycolysis in cancer cells issues from recent studies suggesting an important role for lactate use by cancer cells as a primary TCA substrate (17,18). Interestingly, recent data suggest that circulating lactate, rather than lactate generated from cancer cells themselves, may serve as the source of this TCA fuel, a finding that may have important implications for metabolic imaging paradigms (17).

Although the metabolic implications of glycolytic modulation are vital, an increasing body of evidence supports a role for lactate, and other metabolites, in conditioning the tumor microenvironment to mitigate antitumor immunity (19). Aerobic glycolysis results in the depletion of glucose and production of lactic acid with concomitant acidification of the tumor microenvironment, which impairs T-cell function and activation. Activated T cells rely on glucose for clonal proliferation and cytokine production. As such, antitumor immunity is inhibited by depriving T cells of this essential substrate (20–24). Similarly, lactate production and acidification have been shown to impair interferon-γ expression in tumor infiltrating T cells and natural killer cells, with resultant inhibition of immunosurveillance and promotion of tumor growth (25). Moreover, lactate has been shown to directly suppress the proliferation and activation of T cells (26,27).

In addition to glucose, the metabolism of other key nutrients becomes similarly dysregulated in cancer. Glutamine, the most abundant amino acid in the plasma, is avidly consumed at higher levels than other amino acids by cancer cells in certain contexts (28). Metabolism of glutamine yields ATP, reducing equivalents, and a carbon source for anabolism, similar to glucose. Glutamine-derived nitrogen may be used for nonessential amino acid and nucleotide biosynthesis (29). Through α-ketoglutarate, glutamine replenishes intermediates of an intact TCA cycle—a process known as anaplerosis—particularly in situations where the flux of glucose-derived carbons into the TCA is limited (30). Ultimately, though, a large proportion of glutamine-derived carbons is released as lactate, with nitrogen being similarly excreted (31). As with glucose, oncogenes and tumor suppressor genes also modulate glutamine metabolism (32), with the Myc oncogene being the most extensively studied in this regard. Myc increases expression of the alanine-serine-cysteine transporter 2 (ASCT2) as well as glutaminase and lactate dehydrogenase-A, leading to increased glutamine consumption and shunting of glucose-derived pyruvate away from the TCA cycle (33). The c-Myc transcription factor suppresses microRNA miR-23a/b, with a resultant increase in glutaminase expression (34). The induction of Myc in glutamine-deprived human cancer cells leads to apoptosis, which can be rescued with pyruvate or oxaloacetate, suggesting the importance of glutamine as an anaplerotic substrate in maintaining the function of the TCA cycle (35). Although fundamental research into glutamine metabolism has demonstrated its central role in cellular metabolism and integration with the more established biology of glucose metabolism, recent data indicate that requirements for glutamine may be heterogeneous and context-dependent, underscoring the importance of further studies to more completely define these variations (36,37).

Recognizing the importance of glucose and glutamine in cancer metabolism, a recent study explored the contribution of these nutrients to cell mass. The consumption of glucose and glutamine surpassed that of the other amino acids in 2 mammalian cell lines that use aerobic glycolysis. However, glucose and glutamine did not contribute to most of the carbon cell mass, with other amino acids accounting for the majority. This discrepancy between glucose and glutamine consumption and incorporation into the cell mass suggests additional uses for these nutrients beyond biosynthetic precursors (28), noting lactate excretion from glucose as described above. As a result, altered glucose and glutamine metabolism represent core features of the dysregulated cellular metabolism that characterizes cancer.

Similar to glucose and glutamine, acetate metabolism may become dysregulated in cancer. After conversion to acetyl-CoA, acetate can contribute to the TCA cycle for energy production as has been observed in the myocardium as well as cancers in certain contexts (38,39). Alternatively, acetate can be used in biosynthetic pathways, most notably to synthesize fatty acids and lipids (39). Fatty acid synthase, responsible for synthesis of long-chain fatty acids from acetyl-CoA and malonyl-CoA, is overexpressed in several cancers, including breast and prostate (40). These divergent fates of acetate reflect specific cellular metabolic needs—energy versus biosynthesis—and provide robust imaging opportunities. Finally, acetate plays an important role in modulating gene expression through its metabolism to acetyl-CoA, which is also used for histone acetylation (41). Immunohistochemical analyses of human breast, ovarian, and lung cancers demonstrated increased expression of the nucleocytosolic enzyme acetyl-CoA synthetase (ACSS), compared with noncancerous tissues from the same organ. This enzyme plays a prominent role in histone acetylation through the synthesis of acetyl-CoA from acetate (42).

## IMAGING GLUCOSE METABOLISM

PET imaging with ^18^F-FDG dominates clinical molecular imaging and has found numerous applications in oncologic imaging, including cancer detection, monitoring response to therapy, and prognosis (43,44). Nonetheless, ^18^F-FDG PET has certain inherent limitations. ^18^F-FDG directly probes only a single aspect of cellular metabolism: the delivery of the glucose analog and its phosphorylation. The complexities of metabolism beyond these early steps in glycolysis are not directly measured by ^18^F-FDG. Moreover, in routine clinical practice, an SUV—the concentration of radioactivity in a region of interest normalized to injected dose and body weight—obtained from a static image is routinely reported and compared with prior studies to assess treatment response (43). Blood perfusion, vascular volume of tumor, and nonspecific radiotracer uptake, among other factors, may all affect radiotracer uptake in lesions, confounding the ability of a single SUV to adequately describe the glycolytic state of a tumor (45). By examining only static images, valuable kinetic information is lost that could further characterize important aspects of tumor biology (46). Moreover, the assumptions underlying the interpretation of the biology of ^18^F-FDG as a surrogate for native glucose are complex, as illustrated below in the discussion of the proportionality constant between glucose and ^18^F-FDG.

Analyses of dynamic imaging can yield valuable information beyond the SUV obtained from a static image. Estimated rate parameters (including *K*_1_, the transport rate constant from blood to tissue; *k*_2_, the reverse of *K*_1_; *k*_3_, the phosphorylation constant; and *K*_*i*_*,* representing overall ^18^F-FDG flux) can better characterize ^18^F-FDG uptake and parse the contributions from delivery versus phosphorylation. The correlation of ^18^F-FDG kinetics with tumor blood flow, as estimated by ^15^O-water PET, has been extensively studied in patients with locally advanced breast cancer undergoing neoadjuvant chemotherapy. A low ratio of glucose metabolism (as estimated by ^18^F-FDG flux) relative to glucose delivery (as estimated by ^18^F-FDG transport, *K*_1_, and blood flow) after neoadjuvant chemotherapy was associated with a favorable treatment response (47). In a larger follow-up study, a multivariate analysis that accounted for known clinical and pathologic prognostic factors demonstrated that changes in ^18^F-FDG kinetic parameters and blood flow were more predictive of disease-free survival and overall survival (48). The biologic implications of mismatched perfusion and metabolism have been most thoroughly explored in the assessment of myocardial viability, which has direct clinical implications. Myocardium with decreased perfusion, but preserved metabolism (a perfusion–metabolism mismatch), remains viable and amenable to revascularization (49). These findings demonstrate the complexity of ^18^F-FDG imaging analysis, particularly with regard to the static images used clinically. These complexities underscore the need to better characterize the biology downstream from hexokinase flux, including the use of alternative metabolic fuels as imaging biomarkers.

As a glucose analog, ^18^F-FDG does not precisely recapitulate glucose metabolism. The rate of transport, phosphorylation, and volume of distribution of ^18^F-FDG differ from that of glucose. As such, glucose consumption cannot be directly calculated from ^18^F-FDG kinetics without a proportionality constant relating the rate of ^18^F-FDG metabolism to that of glucose. This constant, known as the ^18^F-FDG lumped constant (LCFDG), includes both the Michaelis constant (K_m_) and the maximal velocity (V_max_) for ^18^F-FDG and glucose, the ratio of their volume of distributions, and a term denoting the proportion of glucose that is metabolized after phosphorylation (assumed to equal 1). The metabolic rate of glucose can be calculated by dividing the ^18^F-FDG metabolic rate by this constant. Spence et al. studied 40 patients with malignant gliomas and demonstrated the lumped constant in gliomas to be greater than that of the contralateral normal brain, confounding the characterization of foci of increased ^18^F-FDG uptake in the brain as malignancy in these patients. An increased metabolic rate of ^18^F-FDG may therefore represent an increased rate of glucose metabolism, a greater lumped constant, or the product of the two (50). The metabolic rate of glucose does not directly equal that of ^18^F-FDG. This fundamental difference, often compounded by the analysis of single static images, illustrates the complexity of interpreting the biology of ^18^F-FDG uptake. More importantly, glycolysis and metabolism extend beyond the activity of hexokinase, providing additional opportunities for therapy and probe development.

Several studies have investigated the mechanistic basis for elevated ^18^F-FDG uptake in cancer. Molecular heterogeneity among tumors has been suggested as a possible source of such variation. In a study of genetically engineered mice with oncogene-driven mammary tumors, significant differences in ^18^F-FDG uptake were seen among tumors of different genotypes, suggesting an underlying molecular basis. Mechanistically, hexokinase-2 protein levels were significantly correlated with mean ^18^F-FDG uptake across all tumors, with additional analysis demonstrating hexokinase-2 was as an independent predictor of ^18^F-FDG uptake after accounting for other variables. By comparison, Glut1 protein demonstrated a modest association with the capacity for uptake that was not significant (51). In malignancies with gluconeogenesis and altered glycogen metabolism, phosphatases may in part account for variable ^18^F-FDG uptake. For example, a recent study demonstrated that expression of fructose-1,6-bisphosphatase, the rate-limiting enzyme in gluconeogenesis and a tumor suppressor gene, inversely correlated with ^18^F-FDG avidity in hepatocellular carcinoma (52). Nevertheless, a preponderance of the data suggests that hexokinase is the rate-limiting step for ^18^F-FDG uptake in most cancer types.

Given the clinical availability and acceptance of ^18^F-FDG PET, research and interest in ^18^F-FDG PET remain strong and well funded. However, the inherent limitations described above continue to be important considerations. ^18^F-FDG PET probes early steps in glycolysis, and the ultimate fate of glucose cannot be queried with ^18^F-FDG. As a result, the complete characterization of glucose metabolism requires investigation beyond ^18^F-FDG flux through hexokinase. Moreover, a complete characterization of cancer metabolism requires the investigation of other pathways and alternative fuels that become similarly dysregulated. A more comprehensive approach to the imaging of cancer metabolism must include new radiotracers and additional modalities that offer complementary measurements.

### MR Methods to Measure Lactate and Other Relevant Substrates

#### MRS and MRSI Approaches

MRS and MRSI are well established and effective techniques for measuring tumor metabolism in vivo (53) by leveraging the MR visibility of nuclei with nonzero spin (odd number of protons or neutrons, or both) (54). MRS enables the noninvasive detection of individual endogenous metabolites or infused metabolites labeled with a stable isotope in tumors without exposure to ionizing radiation (55). Given the low sensitivity of nuclear magnetic resonance–visible heteronuclei, including ^13^C and ^31^P, the application of MRS and MRSI has focused on the detection of protons (homonuclei) in metabolites of interest. An MRS spectrum depicts signal detected from these protons after excitation with a radiofrequency pulse (54). MRS has been most extensively studied in brain tumors, with peaks of *N*-acetylaspartate (a marker of neuronal tissue), choline (a cell membrane marker), creatine (a marker of energy metabolism), and lactate peaks used to characterize tumors (56). Notably, brain tumors have an elevated ratio of choline to *N*-acetylaspartate (57). With regard to glucose metabolism, elevated lipid plus lactate levels were seen in hyperperfused regions of high-grade gliomas compared with hyperperfused regions of low-grade gliomas, with perfusion assessed by arterial spin labeling (58). In a study of breast cancer patients undergoing neoadjuvant chemotherapy, changes in choline measured using MRS correlated with SUV_max_ on ^18^F-FDG PET, suggesting MRS as a possible alternative to ^18^F-FDG PET for serial imaging (59). More recently, advances in indirect ^13^C MRS hold the potential to enable the measurement of ^13^C-labeled substrates in patients (60,61). Despite this utility, the clinical applications of MRS and MRSI remain limited by suboptimal sensitivity, as well as convoluted spectra. In the case of proton MRS, overlapping resonances often require complex editing algorithms (54). To distinguish the lactate resonance from that of lipids in ^1^H-MRS, several techniques have been developed (62). These limitations have motivated the development of alternative MRS- or MRSI-based strategies to measure tumor metabolism, including hyperpolarization of NMR-sensitive nuclei using dynamic nuclear polarization, and CEST.

#### CEST to Image Lactate and Glucose

CEST is an MR-based technique that enables the indirect detection of exogenous or endogenous compounds with exchangeable protons. Unlike conventional MRI, CEST does not require exogenous contrast in high concentrations. In this technique, protons of a compound of interest are selectively saturated with radiofrequency irradiation. The saturated protons are then exchanged with those of bulk water, with a resultant attenuation of the water signal. Normalizing the water saturation signal to the signal before saturation and plotting against saturation frequency yields a Z-spectrum (63). The decreased water signal is proportional to the concentration of the irradiated solute, enabling indirect measurement of the species of interest. Amine (-NH_2_) protons on glutamate and creatine have been imaged, as have hydroxyl (-OH) protons on glycogen, glucose, and lactate, among others (64,65). Lactate CEST has been studied in mouse lymphoma xenografts and skeletal muscle of healthy humans. In the xenograft models, increased lactate CEST signal was seen in the implanted tumor after pyruvate administration (65).

Glucose CEST has also been studied after the infusion of exogenous unlabeled glucose. In mouse xenograft models, glucose CEST showed differences in signal between 2 human breast cancer cell lines (66) and 2 human colorectal tumors (67). Glucose CEST has been translated for clinical application, including studies in glioma patients at 7 T (68) and in head and neck cancer patients at 3 T (69), noting technical advantages of high field strength for separation of saturation frequencies (63). In this regard, glucose CEST offers a promising approach to image glucose metabolism without ionizing radiation. Imaging of numerous other compounds with CEST has been attempted; however, important technical challenges remain to be overcome before clinical translation can be achieved (63).

### Hyperpolarized MRI of Pyruvate

PET detects signal from a radionuclide without the ability to distinguish the parent substrate from downstream metabolites, which may necessitate complex kinetic analysis to capture the relevant biology. In contrast, dynamic nuclear polarization MRSI enhances the sensitivity of detection by hyperpolarizing ^13^C-labeled substrates through the transfer of the spin angular momentum of an electron to the nucleus of interest. The resultant increase in signal-to-noise ratio enables the detection of the parent substrate and its downstream metabolites in real time for the quantitative assessment of metabolic flux (70,71).

Dynamic nuclear polarization MRSI requires the injection of the hyperpolarized ^13^C-labeled metabolite with image acquisition performed on a conventional MRI scanner. Hyperpolarization denotes an artificial, time-limited state of nuclear spin nonequilibrium (71). Technical constraints of low temperature and microwave irradiation for the induction of dynamic nuclear polarization require that the hyperpolarization process take place outside the subject of interest. This process produces a 10^5^ increase in signal enhancement in spin polarization compared with thermal equilibrium. On chemical conversion of the hyperpolarized parent substrate in vivo, hyperpolarized metabolites may form with different chemical shifts, enabling dynamic detection in MR spectra. With sequential imaging, metabolic rates can be quantified (70). However, the hyperpolarized spin state decays to equilibrium over time, with a time constant proportional to the spin-lattice relaxation time, T_1_. As such, the signal available for dynamic imaging is transient typically lasting on the order of 1–2 min (70,71). Thus, the optimal stable isotope for hyperpolarization should fulfill 2 primary requirements: inform important biology and have a T_1_ that is long enough to permit imaging.

Although many metabolites have been hyperpolarized and applied for in vitro and preclinical in vivo studies in cancer, pyruvate has been the most extensively studied because it fulfills these requirements through its combination of favorable physical and biologic properties (72). Given the central role of the conversion of pyruvate to lactate in cancer metabolism (i.e., the Warburg effect), hyperpolarized pyruvate has proved useful in characterizing malignancy in preclinical studies. Unlike imaging with radiotracers, which require only tracer quantities (microdoses), the concentration of injected hyperpolarized substrates is on the order of millimoles and is sufficient to perturb biologic pathways, potentially complicating data interpretation (71,73).

**FIGURE 2.**
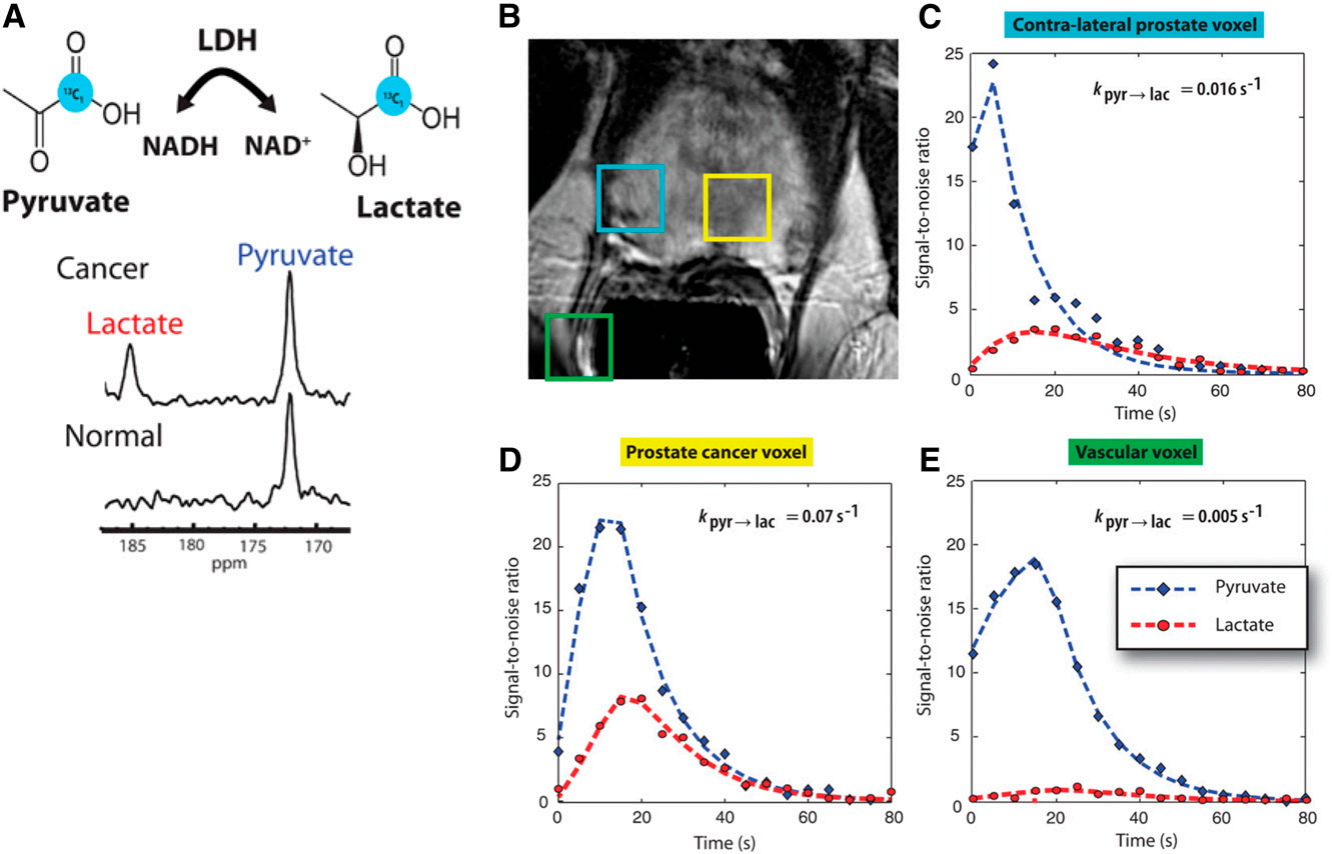
(A, top) Chemical diagram showing metabolism of [1-^13^C]pyruvate to [1-^13^C]lactate catalyzed by lactate dehydrogenase. (A, bottom) Representative ^13^C spectra obtained after injection of hyperpolarized [1-^13^C]pyruvate show increased [1-^13^C]lactate relative to [1-^13^C]pyruvate in prostate cancer compared with normal prostate. (B) T2-weighted MR image from different patient shows findings of prostate cancer (yellow square) and adjacent normal contralateral prostate (turquoise square), as well as a vessel outside prostate (green square). (C–E) Curves fit from 2-dimensional ^13^C dynamic MRSI acquisitions in this patient demonstrate increased flux of [1-^13^C]pyruvate to [1-^13^C]lactate in corresponding regions of prostate cancer (D) compared with normal prostate (C) and vasculature outside prostate (E). (Adapted and reprinted with permission of (75).)

The benefits of dynamic imaging of metabolism without the use of ionizing radiation have enabled translation of this modality into the clinic. In a mouse model of prostate cancer, hyperpolarized lactate and alanine were identified after injection of hyperpolarized pyruvate. Elevated lactate was seen in prostate tumors compared with normal prostate; lactate levels also correlated with histologic tumor grade (74). The clinical application of hyperpolarized imaging of 1-^13^C-pyruvate in humans demonstrated similarly encouraging results. In keeping with the Warburg effect, elevated hyperpolarized lactate-to-pyruvate ratios were seen in biopsy-proven prostate cancers as compared with normal tissue (Fig. 2 (75)).

## IMAGING METABOLIC PATHWAYS BEYOND GLYCOLYSIS

### Imaging of Glutamine Metabolism

#### PET

Increased recognition of the potential importance of glutamine as a metabolic substrate, as described above, has spurred the development of radiolabeled glutamine for imaging. Synthesis of both ^18^F- and ^11^C-labeled glutamine was first reported in 2011 (76,77). As chemically identical compounds, ^11^C-labeled glutamine and unlabeled glutamine share an analogous and complex metabolism. As such, the ^11^C radiolabel is rapidly passed to metabolites and distributed in numerous cellular compartments for biosynthesis, energy production, and excretion. l-[5-^11^C]-glutamine has been studied preclinically in a mouse glioma xenograft and transgenic mice bearing M/tomND spontaneous human mammary tumors (77). Such complexity, combined with a relatively short half-life, will likely confine ^11^C-labeled glutamine to research applications.

**FIGURE 3.**
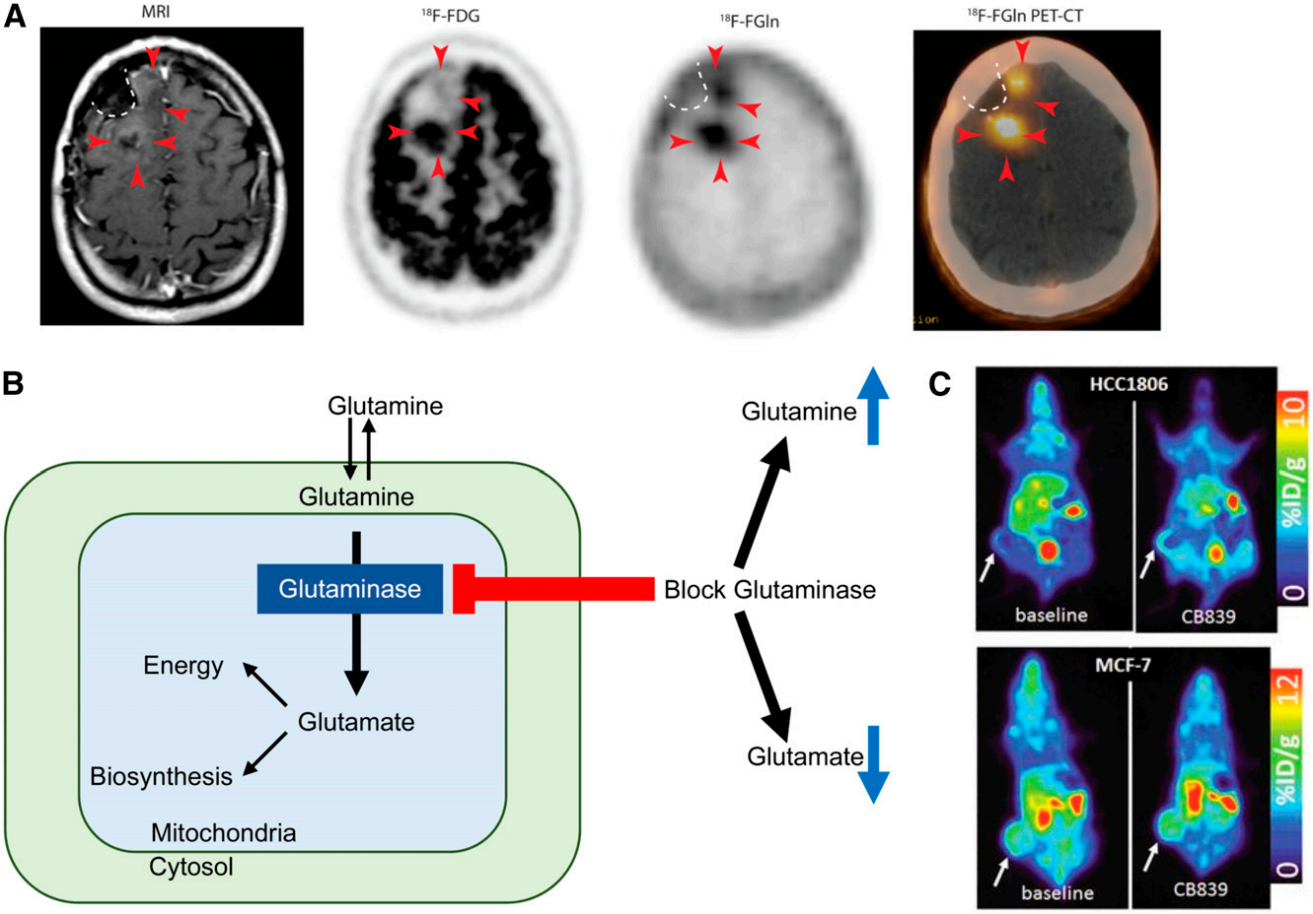
(A) T1-weighted MRI with contrast shows minimal enhancement (arrowheads) along surgical cavity (dotted line) in glioma patient. Corresponding ^18^F-FDG PET image shows uptake in tumor posteriorly but not anteriorly (arrowheads). Corresponding ^18^F-glutamine (Gln) PET image shows tumor uptake both posteriorly and anteriorly. This patient had clinically progressive disease. (Adapted and reprinted with permission of (79).) (B) Schematic of glutamine metabolism and effect of glutaminase inhibitors. With glutaminase inhibition, cellular glutamine increases whereas cellular glutamate decreases. (C, top) ^18^F-glutamine PET images of triple-negative breast cancer xenograft show increased ^18^F-glutamine uptake after glutaminase inhibition reflecting increased glutamine pool size. (C, bottom) In contrast, receptor-positive breast cancer xenograft shows high uptake of ^18^F-glutamine at baseline without increase after glutaminase inhibition reflecting inherently low glutaminase activity. (Adapted and reprinted with permission of (81).)

The addition of a fluorine moiety substantially changes the distribution and metabolism of glutamine, which has enabled translation for human applications. ^18^F-(2*S,*4*R*)4-fluoroglutamine (^18^F-Gln) shares the same transporters as native glutamine but is metabolized to a limited degree. ^18^F-Gln has demonstrated uptake in rats bearing 9L tumor xenografts, as well as in genetically engineered mice with conditional myc gene expression (78). In humans, ^18^F-Gln has been studied in a range of cancers, including glioma, pancreas, and breast (79,80). In 3 glioma patients imaged with clinical disease progression, tumors demonstrated increased ^18^F-Gln uptake. Minimal or no ^18^F-Gln uptake was seen in the 3 patients with stable disease. In contrast to ^18^F-FDG, which demonstrates high background brain uptake, ^18^F-Gln has only minimal uptake in normal brain. These promising early results suggest the utility of ^18^F-Gln in identifying glioma patients at risk of progression (Fig. 3A (79)).

As a minimally metabolized glutamine analog that shares the same transporters as native glutamine, ^18^F-Gln uptake has been proposed as a measure of cellular glutamine pool size. In triple-negative breast cancer tumor extracts with inherently high glutamine use, ^1^H MRS demonstrated a relatively small cellular glutamine pool size. After inhibition of glutaminase, the first enzyme in the glutaminolytic pathway, glutamine pool size increased. Conversely, a large glutamine pool size was observed in estrogen receptor–positive tumor extracts with low glutamine use, without a change in pool size after glutaminase inhibition. ^18^F-Gln PET imaging of tumor xenografts underscored these findings, with tumor-to-blood ratios, an approximation of ^18^F-Gln distribution volume, demonstrating concordant results (Figs. 3B and 3C (81)). Kinetic analysis of dynamic images in these same tumor models demonstrated largely reversible uptake of ^18^F-Gln and confirmed ^18^F-Gln distribution volume as a marker of glutamine pool size (82). This work provides a theoretical framework for image interpretation of ^18^F-Gln, which differs greatly from the analysis of ^18^F-FDG, which is trapped. Further studies are required to ensure appropriate image analysis with consideration of tracer pharmacokinetics. Estimation of changes in pool size with ^18^F-Gln provides the ability to infer tumor glutaminolysis in vivo, suggesting its use as a biomarker to select patients for glutaminase therapy. Changes in pool size after glutaminase therapy can provide a measure of pharmacodynamic response to targeted glutaminase therapy. Given the prevalence of glutamine dysregulation in certain cancers, PET imaging with ^18^F-Gln may have broad application beyond a targeted pairing with glutaminase inhibitors.

Whereas imaging with ^18^F-Gln has just reached human patients in early clinical trials, the Food and Drug Administration approved the use of synthetic amino acid anti-1-amino-3-^18^F-fluorocyclobutane-1-carboxylic acid (FACBC) for the detection of recurrent prostate cancer in 2016 (83). This synthetic amino acid with a 4-carbon ring (84) shares transporters with natural amino acids, most notably the alanine-serine-cysteine transporter 2 (ASCT2) (85). Anti-1-amino-3-^18^F-fluorocyclobutane-1-carboxylic acid enables the detection of persistent disease in men with biochemically recurrent of prostate cancer, leveraging the established dysregulation of amino acid use in these tumors (86).

In like manner to ^18^F-labeled glutamine, imaging with ^18^F-labeled glutamate analogs has advanced into early clinical trials. (4*S*)-4-(3-^18^F-fluoropropyl)-l-glutamate demonstrated transport through the cystine/glutamate exchanger system x_c_^−^. This transporter, involved in glutathione biosynthesis and regulation of reactive oxygen species, has high levels of expression in several tumors. As such, this transporter makes an attractive target for tumor imaging (87). In humans, uptake of (4*S*)-4-(3-^18^F-fluoropropyl)-l-glutamate in breast and non–small lung cancer correlated with expression of the x_c_^−^ transporter by immunohistochemistry (88). However, given the subcellular localization of glutamate in the cytosol for glutathione biosynthesis and glutamate in the mitochondria after formation from glutamine via glutaminase (89), (4*S*)-4-(3-^18^F-fluoropropyl)-l-glutamate appears limited in its ability to fully characterize glutamine or glutamate metabolism in malignancy and may be more effective as a biomarker of free radical regulation.

#### Hyperpolarized MRI

Hyperpolarization of 5-^13^C-glutamine has been performed, with clinical translation hampered by a short T_1_ and a limited polarization efficiency. Early work demonstrated the ability to image the conversion of hyperpolarized glutamine to glutamate in human hepatocellular carcinoma cells (90) and human glioma cells. A deuterated glutamine was hyperpolarized in the latter experiment, more than doubling the T_1_ (33 s vs. 15 s in the undeuterated compound) (91). More recently, dynamic nuclear polarization of 5-^13^C-glutamine has been translated for in vivo MRS imaging in rats. Metabolism of the parent substrate to its metabolite glutamate was detected in the rat hepatic tumor but not in normal liver (92). In addition, 1-^13^C-glutamate has been successfully hyperpolarized, enabling the unique potential to measure flux from glutamate to α-ketoglutarate in the TCA cycle (93). With continued technical innovation, hyperpolarization of glutamine or glutamate, as well as other metabolites, may hold the potential for human translation (71).

#### CEST

CEST imaging of glutamate has been successfully translated into humans, demonstrating the capability to detect temporal lobe epilepsy in patients without a detectable lesion on conventional MRI. Glutamate CEST identified the laterality of a seizure focus in 4 of 4 patients with epilepsy (94). Increased glutamate in seizure foci marks mitochondrial and metabolic injury, which may represent the result of and cause of a seizure in a self-propagating process (95). In like manner to PET, glutamine CEST may have applications in oncologic imaging as a measure of tumoral glutaminolysis based on glutamate pool size.

### Imaging Acetate Metabolism

**FIGURE 4.**
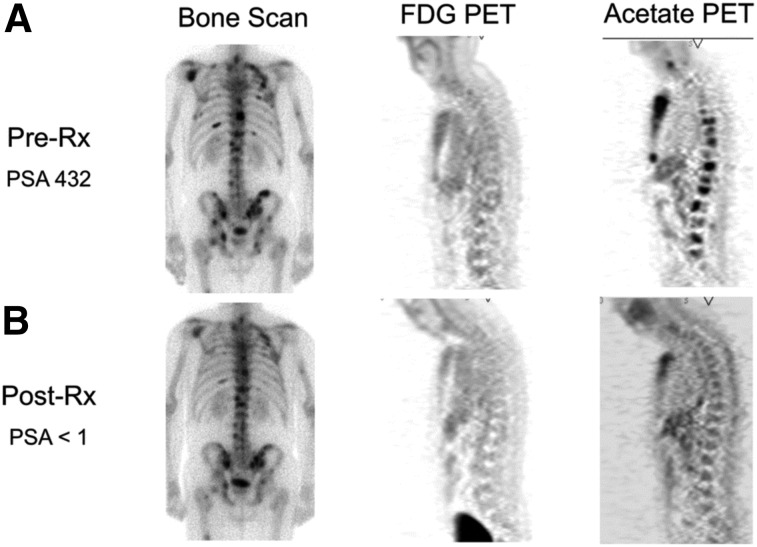
Comparison of bone scan, ^18^F-FDG PET, and ^11^C-acetate PET before (A) and after (B) androgen deprivation therapy in patient with osseous metastases from prostate cancer. ^11^C-acetate demonstrates response to treatment. Bone scan does not demonstrate significant change, and ^18^F-FDG PET fails to detect osseous metastases at either time point. PSA = prostate-specific antigen level. (Reprinted with permission of (100).)

Imaging opportunities with acetate parallel its metabolic fate, holding the potential to provide measures of TCA metabolism. Indeed, studies dating back into the 1980s demonstrated that radiolabeled acetate metabolism can estimate TCA cycle flux in the myocardium as a measure of myocardial energy metabolism that is proportional to oxygen consumption (96). Metabolism of acetate is measured as the clearance rate from the myocardium, which is indicative of labeled-acetate metabolism as the radiolabel passes to downstream TCA molecules and eventually to radiolabeled CO_2_, which is cleared rapidly from tissues (38). As opposed to cardiac metabolism, which uses acetate almost entirely for energy production, cancer cells also metabolize acetate for lipid synthesis (97), as a key component in membrane synthesis that is required for the proliferative phenotype (98). Unlike acetate energy metabolism, acetate incorporation into lipids and other molecules used for biogenesis results in trapping of the ^11^C label, which can be measured as a trapping flux constant (*K*_*i*_) or by static uptake measures late after injection (99). ^11^C-acetate has been extensively studied in prostate cancer for primary staging, assessing regional lymph node involvement and distant metastatic disease, and in biochemical recurrence (98). A pilot study of ^11^C-acetate in prostate cancer with bone metastases demonstrated a correlation between assessment of tumor response with ^11^C-acetate and clinical response, suggesting the utility of this radiotracer for treatment response (Fig. 4 (100)). ^11^C-acetate has been studied in other malignancies, notably bladder and renal cell carcinoma given the lack of urinary excretion, as well as hepatocellular carcinoma (101). These data underscore the potential role of acetate as a marker of cancer metabolism as ^11^C-acetate holds great promise as a radiotracer indicating the balance of energy metabolism and biogenesis in the TCA cycle. The ability to measure both energy metabolism and biosynthetic flux in cancer using ^11^C-acetate has been a challenge but may be possible with alternative approaches (102), or possibly with the combination of PET and dynamic nuclear polarization MRSI methods, which can track the biochemical fate of a labeled substrate through the detection of its metabolites (103).

## CONCLUSION

In this era of precision medicine, targeted therapies have transformed the treatment of many malignancies. The translation of targeted imaging modalities to predict and monitor the response to new therapies has lagged behind these therapeutic advances. The ability to optimize these precision medicine paradigms requires the development of new imaging biomarkers. Metabolic imaging with novel radiotracers and MRI-based imaging agents has shown early promise primarily in the preclinical setting. These emerging approaches to imaging cancer metabolism are now primed for clinical application. Given the unique and synergistic capabilities of these modalities, combined approaches using both PET and MR are likely to better characterize tumor biology than either approach alone. Instead of relying on biopsies for tumor characterization and anatomic measurements for response assessment, the integration of these novel approaches offer the potential for a noninvasive and targeted characterization of malignancy. Such information will better inform treatment and ultimately improve patient outcomes.

## DISCLOSURE

This work was supported by KL2TR001879, R01CA211337, Komen SAC130060, DP5-OD021391, and R33CA225310. No other potential conflict of interest relevant to this article was reported.
